# About 3D Printability of Thermoplastic Collagen for Biomedical Applications

**DOI:** 10.3390/bioengineering9120780

**Published:** 2022-12-07

**Authors:** Marina Passos, Sergej Zankovic, Graça Minas, Enno Klüver, Marit Baltzer, Hagen Schmal, Michael Seidenstuecker

**Affiliations:** 1 G.E.R.N. Center of Tissue Replacement, Regeneration & Neogenesis, Department of Orthopedics and Trauma Surgery, Medical Center-Albert-Ludwigs-University of Freiburg, Faculty of Medicine, Albert-Ludwigs-University of Freiburg, Hugstetter Straße 55, 79106 Freiburg, Germany; 2 Center for Micro Electromechanical Systems (CMEMS-UMinho), University of Minho Campus de Azurém, 4800-058 Guimarães, Portugal; 3 LABBELS-Associate Laboratory, University of Minho Campus de Gualtar, 4710-057 Braga, Portugal; 4 FILK Freiberg Institute gGmbH, Meissner Ring 1-5, 09599 Freiberg, Germany

**Keywords:** bone tissue engineering, 3D printing, TC, rheological analyze, mechanical properties

## Abstract

With more than 1.5 million total knee and hip implants placed each year, there is an urgent need for a drug delivery system that can effectively support the repair of bone infections. Scaffolds made of natural biopolymers are widely used for this purpose due to their biocompatibility, biodegradability, and suitable mechanical properties. However, the poor processability is a bottleneck, as highly customizable scaffolds are desired. The aim of the present research is to develop a scaffold made of thermoplastic collagen (TC) using 3D printing technology. The viscosity of the material was measured using a rheometer. A 3D bioplotter was used to fabricate the scaffolds out of TC. The mechanical properties of the TC scaffolds were performed using tension/compression testing on a Zwick/Roell universal testing machine. TC shows better compressibility with increasing temperature and a decrease in dynamic viscosity (η), storage modulus (G′), and loss modulus (G″). The compressive strength of the TC scaffolds was between 3–10 MPa, depending on the geometry (cylinder or cuboid, with different infills). We have demonstrated for the first time that TC can be used to fabricate porous scaffolds by 3D printing in various geometries.

## 1. Introduction

Although prophylaxis and aseptic surgical techniques have improved, inflammatory musculoskeletal disease has been around since the dawn of mankind. It remains a complex problem to manage, with over 1.5 million total knee and hip replacements performed annually [1,2]. This fact is directly related to the increase in the population’s average age. According to the Statistical Office of the European Union (Eurostat), the population of the European Union (EU) continues to age, with a visible increase over the past 20 years [3]. Almost one in three people in the EU are 65 years or older. In 2017, the percentage was 29.9%, increasing to 34.8% by 2020 [3,4]. According to Eurostat, the percentage of elderly people in 2100 will be 57%, reflecting a higher increase in Poland (63%), followed by Italy, Malta, and Finland (all 62%), as well as Croatia (61%) [5]. There is an urgent need for a drug-loaded delivery system that can effectively deliver antibacterial drugs to the injured area.

To avoid the complications associated with autografts, allografts, and xenografts, bone substitutes, called bone scaffolds, have been developed. Alonzo et al. [6] defined tissue engineering as follows: “Tissue engineering has become an alternative strategy targeting bone repair through the use of a synergistic combination of biomaterial scaffolds, cells, and signalling molecules/growth factors to induce the formation of new bone tissues by eliminating the risks associated with autografts”. In agreement with Chen et al. [7], there are five key requirements for an optimal bone scaffold: high porosity and connectivity, excellent mechanical properties, degradability, and biocompatibility. In this context, a wide range of natural biomaterials are used, characterized by their biocompatibility, biodegradability, and non-toxicity compared to non-natural materials. The chitosan (CS) and Alginate (Alg) fulfill four of the five key requirements for an optimal bone scaffold. However, these materials have low mechanical strength [8,9,10]. Several studies have been conducted to combat this disadvantage. He et al. [9] and Gupta et al. [11] have shown in their studies that the mechanical properties improve when hydroxyapatite is added to these materials. However, other disadvantages such as poor reproducibility for chitosan and low cell adhesion for alginate, remain a problem [8,9,10,11]. Therefore, to overcome this problem, in the present study, another form of collagen, the TC was used as xerogel [12]. 

Material selection and manufacturing techniques for the scaffold play a crucial role in choosing the optimal device for a drug—loaded delivery system. Certainly, advanced processing techniques are required to facilitate the fabrication of highly customizable scaffolds. Conventional methods for fabricating porous 3D scaffolds, such as particle leaching, foaming, or freeze-drying, have several drawbacks related to architectural control and internal pore interconnectivity [13]. Thus, 3D printing can overcome these limitations and is a promising technology for the future. This technology allows fabricating scaffolds with adjustable mechanical properties and benefits the ability to control the cellular response with the addition of cells, bioactive molecules and/or drugs [14]. One of the most commonly used 3D printing processes is selective laser sintering (SLS). In this method, a powder is selectively melted with a laser. The main limitation of this technique is the structure’s size, which is directly related to the particle size of the powder [15,16]. The fused deposition modelling (FDM) process is another of these 3D printing processes. This technique overcomes the main limitation of SLS; however, it is limited to thermoplastic materials (unprocessed) [13,14,15,16,17]. Another technique is 3D plotting, which is an integral part of 3D printing research. With this method, it is possible to use thermoplastic materials and create layer-by-layer customizable geometries, through 3D digital models created with computer-aided design (CAD) software [13,14]. The main objective of this study is to explore the dynamic viscosity of TC and test its printability, using a 3D-Bioplotter. In addition, in contrast to the previous work [18], 3D structures should be printed and blocks should not be the only things extruded. The blocks would be rather unfavorable with regard to possible applications because there would be no possibility of cell migration, and in addition, only a superficial release would be possible for applications such as drug delivery. This will be further addressed in this work and 3D structures in different geometries (square, round geometries for the outer shape with lines, honeycombs as inner shapes) with storage rotation of the layers will be produced. The alternating shapes also create defined macro pores or an open cell structure.

## 2. Materials and Methods

### 2.1. Materials

TC with a particle size of <250 µm (TC4261) was produced from bovine hides at FILK Freiberg Institute gGmbH (Freiberg, Germany). Briefly, raw hides were dehaired in a standard lime process with sodium sulfide at pH 12. The dehaired pelts were dried at 80 °C in a rack dryer to denature them. The dry hides were ground to a fine powder in a granulator (SM2000, Retsch, Haan, Germany) and turbo mill (Görgens Engineering GmbH, Dormagen, Germany) and subsequently sieved. Details can be found in [12]. Glycerine was purchased from Sigma (Sigma-Aldrich GmbH, Steinheim, Germany). The printing ink was prepared at a concentration of 25:73:2%, TC:water:glycerine using TC powder manually mixed with water and glycerine at a density of 1.26 g/cm^3^. The mixture of water and glycerine was first kept at 70 °C for 5 min in a safety heating plate (IKA-RCT basic IKAMAG^®^, IKA-Werke GmbH & Co. KG, Staufen, Germany). Glycerine and water were then added to the TC. The mixture was homogenized by using an IKA^®^ EUROSTAR 20 high-speed digital stirrer with a three-bladed propeller shaft (IKA^®^-Werke GmbH & Co. KG, Staufen, Germany) at 500 rpm for 7 min. The sol was kept in the refrigerator for two hours until it became solid. The solid material was cut into small cylinders by using a 6 mm diameter punch (Peddinghaus, Schwelm, Germany) and placed in the high-temperature print head of the 3D-bioplotter.

### 2.2. Methods

#### 2.2.1. Rheology

The dynamic rheology of TC was performed using mixtures of 25:73:2% TC:water:glycerine. These measurements were performed using an Anton Paar Physica MCR 301 rheometer (Anton Paar GmbH, Graz, Austria) according to DIN 3219 norm. It consists of a parallel plate geometry with a diameter of 50 mm (PP50). The storage modulus (G′), loss modulus (G″), loss tangent (tan δ = G″/G′), and dynamic viscosity (η) as a function of temperature (°C) were determined continuously during the test. The temperature was decreased from 119 to 98 °C with a ramp rate of 2 °C/min. The shear rate used, was 0.1 s^−1^. Data from all rheological measurements were analyzed using the supporting software Rheoplus 32 v3.40 (Anton Paar GmbH, Graz, Austria).

#### 2.2.2. Effect of Dynamic Viscosity on Pressure

**Figure 1:**
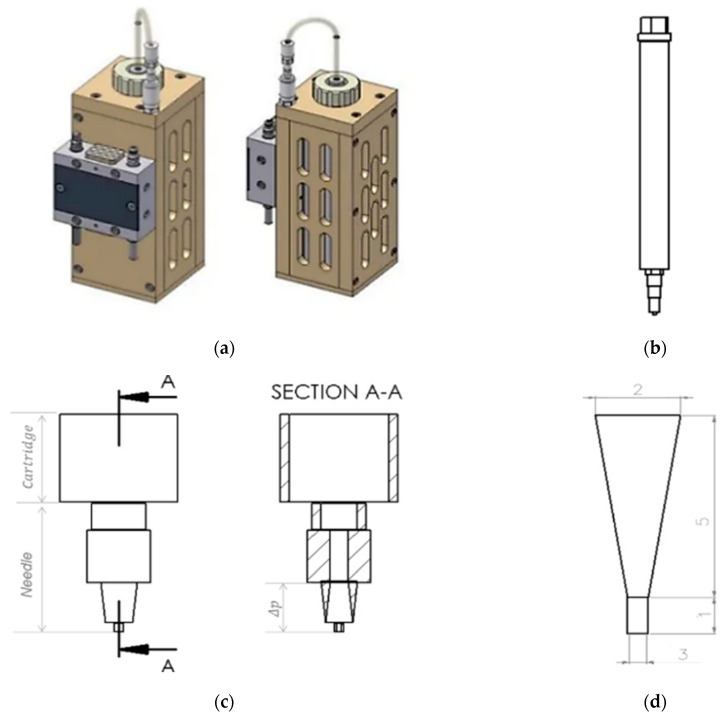
Picture of a high-temperature-viscous dispensing head of the 3D-Bioplotter, front view and side view (**a**); Drawing of a stainless steel cartridge, with luer-lock adapter, cartridge cap, and Fastflow needle from EnvisionTEC (**b**); Drawing of the lower part of the cartridge and the needle, front view and inside view (section A-A) (**c**); Drawing of the lower part of the needle, considering the pressure drop in the upper part of the static needle. Thus, the length of the lower part of the needle is equal to 6 mm and the diameter of the lower part of the needle is 1.15 mm (**d**) From SolidWorks^®^ 2020, courtesy of EnvisionTEC GmbH, Gladbeck, Germany.

To evaluate the effect of dynamic viscosity on pressure, a 25:73:2% mixture of TC, water and glycerine was used (Section 2.1). For this analysis, Hagen Poiseuille’s law was used assuming TC is a non-compressible fluid. The dynamic viscosity (η) of the material, the radius at the fourth power (r^4^), the length of the needle (L) used to extrude the material, the volumetric flow rate of the extruded material (Q), and the pressure drop between the needle tip and the inside of the cartridge (Δp_needle tip_) were used for the analysis (see in the Equation (1) [19]. For this purpose, the dynamic viscosity of the TC mixture was determined previously (Section 2.2.1) using the Anton Paar-Physica MCR 301 rheometer (Anton Paar GmbH, Graz, Austria). The volumetric flow rate of the extruded material (Q) was determined according to Duty et al. [19]. In their study on the optimal conditions for successful 3D printing, they showed that Q can be calculated using the relationship between the diameter (d) of the needle and the extrusion speed (v) used, as shown in Equation (2). This equation was used assuming that the diameter of the printed strand is equal to the diameter of the needle. A Burg-Wächter PS 7215 digital caliper (Burg-Wächter; Wetter-Volmarstein, Germany) was used to measure the length and diameter of the needle. The needle model was designed using SolidWorks^®^ 2020 CAD software (SolidWorks, SolidWorks Corporation, Concord, MA, USA). Figure 1 shows the cartridge design of the high-temperature viscous dispensing head of the 3D-bioplotter and the Fastflow needle from EnvisionTEC, with an inner diameter of 300 µm used for extrusion.

The mathematical equation relating pressure and dynamic viscosity is described as follows:

Δp = (Q·8·η·L)/(π·r^4^) (1)

The volumetric flow rate of the extruded material (Q), can be determined by the following mathematical equation:

Q = π d^2^·v/4 (2)

### 2.3. Manufacturing of the TC Scaffold

TC scaffolds were fabricated using a 3D-Bioplotter (3D-Bioplotter^®^ Manufacturer Series, EnvisionTEC, Gladbeck, Germany). For this purpose, the TC granules were previously stored in the refrigerator for two hours. It was then transferred to the supply cartridge of the high-temperature viscous dispensing head of the 3D-Bioplotter. TC was heated to 90 °C in the printer cartridge for 40 min and extruded at a pressure of 0.9 bar and a speed of 40 mm/s. The Plexiglas plate was cooled to 17 °C using a Peltier-element to ensure that the printed first layer had the optimal geometry. Fastflow needles from EnvisionTEC with an inner diameter of 300 and 400 µm and a needle offset of 0.4 mm were used for printing. The pre-flow and post-flow delay were 0.09 and −0.01, respectively. The waiting time between layers was 120 s. It is important that cooling occurs between each layer. Four different geometries were printed: square base with line fill; square base with honeycomb fill; round base with line fill and round base with honeycomb fill. The scaffolds were printed in 18 layers. The scaffolds with line filling were printed with a rotation of 90° in each layer. For the scaffolds with honeycomb filling, the layers were not rotated. Each geometry was printed 15 times. The parameters for printing a TC scaffold were summarized in Table 1. All samples were dried with a lyophilizer (FreeZone 2.5 plus, Labconco, Kansas City, MO, USA) for 24 h.

### 2.4. Characterization of the TC Scaffold

#### 2.4.1. Dimensions and Weight

Characterization of the size and weight of the TC scaffolds was performed on 15 different samples. A Practum^®^ analytical balance (Sartorius Lab Instruments GmbH & Co. KG, Göttingen, Germany) was used to determine the weight of the scaffolds. The dimensions of the scaffolds were measured with a Burg-Wächter PS 7215 digital caliper (Burg-Wächter; Wetter-Volmarstein, Germany). The KEYENCE VK-X210 3D scanning microscope (Keyence, Osaka, Japan) was used to measure pore size and strand widths and to capture images required for macroporosity analysis. The porosity of the samples was determined based on the ratio between the pore area and the total area.

#### 2.4.2. Surface Roughness

The strand width and surface roughness (Sa) of the extruded strands were analyzed using the VKX-210 3D laser microscope (KEYENCE Corporation, Osaka, Japan). All images were taken with a Nikon lens (Nikon Inc., Minato, Japan) at 20× magnification (equivalent to 400× in the microscope). Three samples were measured at five different locations.

#### 2.4.3. Microstructure by ESEM

To analyze the microstructure of the TC scaffolds, images were acquired using ESEM FEI QUANTA 250 FEG (FEI, Hilsboro, OR, USA) with an acceleration voltage of 5 kV and a secondary electron detector. For this purpose, the samples were cut in the middle with a razor blade (Apollo Herckenrath GmbH & Co., Solingen, Germany), and glued to the sample pin holder with a double-sided carbon conductive pad (Plano GmbH, Wetzlar, Germany). One specimen of each geometry was analyzed.

#### 2.4.4. Mechanical Testing

The tensile-compression test was performed in accordance with DIN EN ISO 604-2003 on 5 different specimens in the Zwick/Roell Z005 universal testing machine (Zwick/Roell, Ulm, Germany). The compression measurement was displacement-controlled at 1 mm/s up to a maximum force of 2000 N.

### 2.5. Statistics

All data in this paper are presented as mean ± standard deviation. All statistical analyses were performed using Origin 2021 Professional SR1 (OriginLab, Northampton, MA, USA). ANOVA was performed at a significance level of *p* < 0.05. Normal distribution was examined by using the Kolomogorov-Smirnov test.

## 3. Results

### 3.1. Viscosity

#### 3.1.1. Rheology

**Figure 2:**
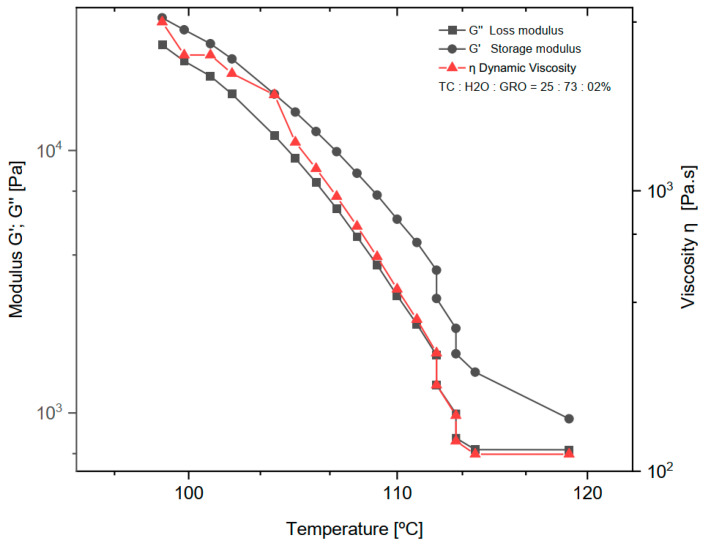
Dynamic viscosity (η), loss modulus (G″) and storage modulus (G′) of the mixture of 25:73:2% of TC:water:glycerine. The normal distribution of the model was verified for each group by the Kolmogorov-Smirnov test. The adjusted R-square (R—sq (adj)) for the dynamic viscosity (η), loss modulus (G″) and storage modulus (G′) was: 0.894, 0.902, 0.937.

The dynamic rheological experiments were carried out at a fixed frequency of 1 Hz for the mixtures of 25:73:2% of TC:water:glycerine, respectively. The viscoelastic properties were studied by measuring the dynamic viscosity (η), storage modulus (G′), and loss modulus (G″) at a temperature decreasing from 119 to 98 °C with a ramp rate of 2 °C/min (See in Figure 2). Below 98 °C the measurement was no longer possible because the shear forces exceeded the maximum of the sensor installed in the rheometer.

#### 3.1.2. Effect of Dynamic Viscosity on Pressure While 3D Printing

**Figure 3:**
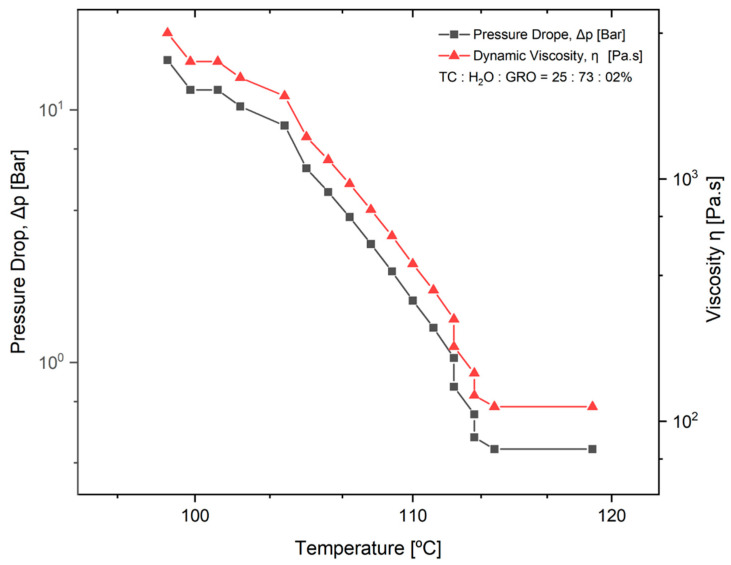
Pressure drop in the tip of the needle (Δp_needle tip_) as a function of temperature and dynamic viscosity of the material. The normal distribution of the model was verified for each group using the Kolmogorov-Smirnov test. The adjusted R-square (R-sq (adj)) for the model was 0.941.

The pressure drop (Δp_needle tip_) during extrusion of the TC was determined. This parameter was determined as a function of the dynamic viscosity of the material and a temperature decrease from 119 °C to 98 °C. The mixtures of 25:73:2% of TC:water:glycerine were used. The results can be seen in the diagram in Figure 3. It can be seen that the higher the viscosity of the material, the greater the pressure drop with decreasing temperature. From 100 to 112 °C, an approximately linear progression of the dynamic viscosity can be observed.

### 3.2. Characterization of the TC Scaffold

#### 3.2.1. Dimensions and Weight

**Figure 4:**
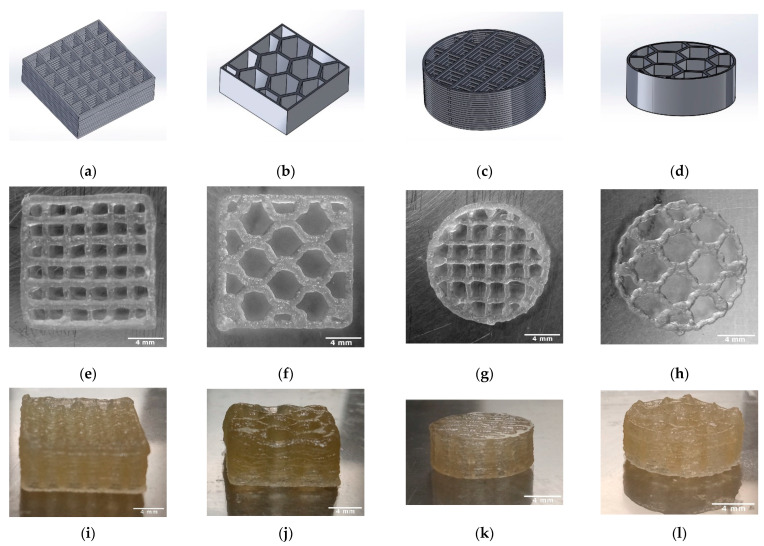
Overview of the 3D printed TC scaffolds with 18 layers; CAD models (SolidWorks^®^ 2020) (**a**–**d**); TC scaffold first layer top view (**e**–**h**); TC scaffold side view (**i**–**l**).

The length, height, weight, strand width, pore size, and macroporosity of the scaffolds were measured to characterize the scaffolds. At least three samples of TC scaffolds were measured once. The samples were approximately square and had a length of 13.63 mm. The scaffolds averaged 5.48 ± 0.03 mm in height and weighed an average of 0.37 ± 0.03 g. The strand widths were 303.66 ± 5.05 µm, and the pore size was 1922.77 ± 10.76 µm. Figure 4 shows the overview of the 3D printing scaffolds. Table 2 shows the dimensions and weights of the TC scaffolds.

#### 3.2.2. Surface Roughness

**Figure 5:**
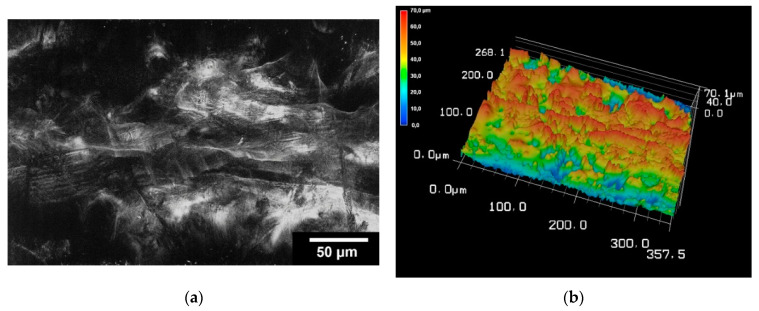
Images of 3D laser scanning of the scaffold surface (**a**); image of 3D reconstruction of the scaffold surface (**b**). The images were taken with KEYENCE VK-X210 3D laserscanning microscope; 200× magnification; scale bar = 4 mm.

The surface roughness was determined for the TC scaffolds. For this purpose, three scaffolds were examined at five different locations. On average, the surface roughness (Sa) of the samples was 2.15 ± 0.43 µm. The surface roughness of the scaffolding can be seen in Figure 5.

#### 3.2.3. Microstructure by ESEM

**Figure 6:**
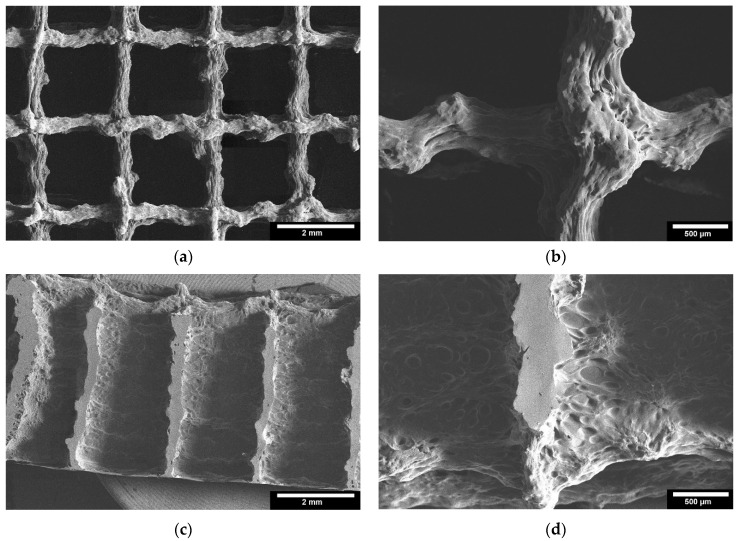
ESEM images of the 3D printed TC scaffolds; 3D printed TC scaffolds top view: (**a**): 22× magnification; (**b**): 65× magnification; 3D printed TC scaffolds cross-section view: (**c**): 22× magnification; (**d**): 65× magnification; images taken with FEI Quanta 250 FEG, 5 kV acceleration voltage; detector for secondary electrons.

Microstructure analysis with ESEM allowed a more detailed examination of the scaffolds. Figure 6 shows images of the TC scaffolds in top view (Figure 6a,b), and in side view of a cryo-broken scaffold (Figure 6c,d). This allowed a cross-section of the scaffold to be examined. The scaffolds did not show any cleavage failure. It can be observed that the layers of the scaffolds are well bonded to each other throughout the joint area.

#### 3.2.4. Mechanical Properties

The compressive strength of the TC scaffolds with mixtures of 25:73:2% of TC:water:glycerine was determined by using the Zwick Universal Testing Machine Z005. The cuboid samples with line infill resulted in a compressive strength of 3.6 ± 0.2 MPa; the cuboid samples with honeycomb infill in 6.9 ± 1.2 MPa, the cylindrical sample with line infill in 3 ± 0.3 MPa and the cylindrical sample with honeycomb infill resulted in a value of 10 ± 1.2 MPa (cf. Table 3).

## 4. Discussion

As expected, water was a successful plasticizer for processing TC at the ink production stage. The addition of glycerol to the sol made the TC ink more flexible and plastic. However, in the study on TC, Klüver et al. [12] stated that “the addition of glycerol significantly decreases the tensile strength (except for a slight increase at low concentrations below 10%), while the elongation increases”. Therefore, the addition of 2% glycerol to TC and water seemed to be the right choice for the present study. The amount of water in the sol was another important consideration. The least amount of water requires high extrusion temperatures. The boiling point of water is 100 °C at 1 atm, and above this value the water evaporates and water loss occurs during extrusion. On the other hand, a high water content can lead to a loss of the most important properties of collagen, such as mechanical properties, osteoconductivity, and biodegradability [20]. In the present work, a water content of 73% and an extrusion temperature of 90 °C were found to be optimal for printing TC. With increasing temperature (°C), a decrease in dynamic viscosity (η), loss modulus (G″), and storage modulus (G′) was observed. The decrease in storage modulus is practically linear, with a range from 25,200 to 725 Pa. For dynamic viscosity and loss modulus, the decrease is linear from 4000 to 115 Pa∙s and 32,000 to 1430 Pa, respectively, until a temperature of about 113 °C was reached. Above this temperature, both the loss modulus and dynamic viscosity do not decrease, and the behaviour becomes practically constant. Comparing the loss modulus and the storage modulus, G′ and G″ decrease at the same rate until G″ reaches its minimum value and assumes a constant behaviour. As expected, according to other literature on thermoplastic materials, the loss modulus curve shows a lower value than the storage modulus over the entire temperature range tested [21].

The pressure drop is an important parameter for good printability of the material, as it was closely related to the needle length and the dynamic viscosity of the material to be plotted. In Figure 3, it can be seen that the pressure drop increases significantly as the viscosity of the material increases, while the temperature decreases. Since the dynamic viscosity determines the printability of the material, the material becomes more printable as the temperature increases. In their studies on the parameters for optimal extrusion, Mishra et al. [22] and Gopi et al. [23] came to similar conclusions as in the present study. According to Duty et al. [19], the material can be successfully deposited if the calculated pressure drop (Δp_needle tip_) is less than the maximum system pressure for a given flow rate (Q). Since the maximum pressure of 0.9 bar was used for the present work, this means that extrusion is no longer possible under conditions where the temperature is below 112 °C. This result does not correspond to the temperature of 90 °C that was used as the standard for printing the scaffolds. This difference can be directly related to the variation in ambient temperature at the 3D-Bioplotter location [24]. Since it was a natural material with high water content, the water content in the sol may decrease and the dynamic viscosity of the material may increase depending on the environment. However, according to a study by Sukindar et al. [25], this problem can be improved by using a small diameter nozzle.

According to Gopi et al. [23], the strand width is an important parameter to be considered for the print quality of the scaffold. The strand width should be as close as possible to the diameter of the nozzle used. If it is much less than the nozzle diameter, this can affect the bonding of the layers, resulting in a loss of the mechanical properties of the scaffold. On the other hand, if the strand width is much larger than the nozzle diameter, it means that the upper layer presses on the lower layers, resulting in the formation of elliptical strands and thus affecting the porosity of the scaffold. In the present study, the TC scaffold strand width of a square structure was 303.66 ± 5.05 µm, and the diameter of the needle used was 300 µm. This result shows that the strand width was quite close to the needle diameter used for extrusion. This is consistent with other studies on scaffolds printed with an EnvisionTec 3D bioplotter [26]. Klüver et al. [18] demonstrated the printability of TC as a solid without pores. But the plotted strands were not stable and showed an increase (39–57%) in the strand diameter directly after printing.

The results of 3D laser scanning microscopy to determine the surface roughness of the TC scaffolds showed slightly lower values of 2.15 ± 0.43 µm compared to our other studies of scaffolds printed with the 3D bioplotter. The studies of Weingartner et al. [26] and Huber et al. [27] on the effect of collagen I coatings of 3D printed PCL scaffolds for bone replacement determined a surface roughness of 4.11 ± 0.27 µm and 5.42 ± 0.82 µm for uncoated specimens and 3.35 ± 0.3 µm and 2.75 ± 0.48 µm for specimens coated with type I collagen. However, these were only coatings with collagen in the micrometre range and not scaffolds of collagen.

In the ESEM images, it was clear from both the top view and the cryo-fracture view that the layers of the scaffolds exhibited good connectivity throughout their composite region. According to Vorndran et al. [28], this good connectivity is favorable to achieve better mechanical properties. In the compression tests, the TC scaffold showed a compressive strength of 10.5 ± 3.5 MPa. This compressive strength refers to the moment at which the material starts to yield. This value is similar to the compressive strength of trabecular bone reported in the literature [29], from 0.52 to 45 MPa for the proximal tibia. Currey et al. [30] reported in their study that cortical bone has a compressive strength in the range of 110–164 MPa, which is a 10–20 fold increase compared to the compressive strength of TC scaffolds. This low strength could be related to the pore size of the scaffold of 1922.77 ± 10.76 µm. Seidenstuecker et al. [31], Han et al. [32], and Ai et al. [22] showed in their studies that the optimum pore size for better mechanical properties is between 160–500 µm. Both honeycomb structures show higher maximum failure load and have higher compressive strength compared to the scaffolds with line filling. We attribute this to the larger area of connectivity between layers. The scaffolds with line filling were printed with a rotation of 90° in each layer, thus they have connectivity only at the line-crossing region. In contrast, the scaffolds with honeycomb filling the layers were not rotated, thus they reveal better connectivity. Slightly higher maximum failure load and compressive strength of cuboid shape compared to the cylindrical shape is due to a larger overall size of the cuboid scaffolds compared to cylindrical structures.

The scaffolds presented here can be used as a bone repair material. Such properties as high macro-porosity and a high level of pore connectivity are favorable for cell migration and tissue ingrowth. Moreover, thermoplastic collagen is expected to be biocompatible and biodegradable. This as well as the swelling behavior of the scaffolds should be addressed in the future work. Another application of the 3D scaffolds from TC could be the drug release carrier.

## 5. Conclusions

The results show for the first time to what extent TC is suitable for 3D printing technology. A major challenge in material extrusion is to determine the range of parameters that will allow proper extrusion of thermoplastics. In this study, scaffolds out of TC were successfully printed in various geometries after determining the appropriate extrusion parameters. Factors such as ambient temperature, pressure drop, platform temperature, and temperature drop in the cartridge and needle have a significant impact on the results. The TC scaffold exhibits a compressive strength similar to that of a trabecular bone, but it is far from reaching the values of cortical bone. Therefore, these scaffolds could be used for future applications such as bone defect repair. Some optimizations of the TC sol, geometry, and structure of the scaffolds could be made in the future to improve the compressive strength.

## Figures and Tables

**Table 1:** General parameters for 3D printing.

Needle Inner Diameter [µm]	Temperature [°C]	Pressure [bar]	Speed [mm/s]	Needle Offset [mm]	Pre-Flow [s]	Post-Flow [s]	Platform Temperature [°C]
300	90	0.9	40	0.3	0.09	−0.01	17

**Table 2:** Size, weight, and strand width of the 3D printed TC scaffolds (n = 15).

Sample	Length [mm]	Height [mm]	Weight [g]	Strand Width [µm]	Pore Size [µm]	Porosity [%]	Nozzle Size [µm]
Cuboid-lines	13.7 ± 0.2	5.5 ± 0.03	0.37 ± 0.03	304 ± 5	1923 ± 11	25.6	300
Cuboid-honeycomb	14.8 ± 0.5	4.4 ± 0.4	0.41 ± 0.06	428 ± 15	2812 ± 47	23.4	400
Cylinder-lines	13.8 ± 0.5	4.5 ± 0.2	0.14 ± 0.007	425 ± 13	1923 ± 11	43.1	400
Cylinder-honeycomb	14.2 ± 0.9	4.8 ± 0.3	0.24 ± 0.03	425 ± 13	2812 ± 47	36.4	400

**Table 3:** Maximum failure load and compressive strength for the different scaffolds (n = 5).

Sample	Maximum Failure Load [N]	Compressive Strength [MPa]
Cuboid-lines	806 ± 43	3.6 ± 0.2
Cuboid-honeycomb	1649 ± 290	6.9 ± 1.2
Cylinder-lines	452 ± 42	3 ± 0.3
Cylinder-honeycomb	1435 ± 172	10 ± 1.2

## Data Availability

The data presented in this article are available on request from the corresponding author.
